# Retraction statement: MicroRNA‐431 inhibits migration and invasion of hepatocellular carcinoma cells by targeting the ZEB1‐mediated epithelial–mesenchymal transition

**DOI:** 10.1002/2211-5463.13333

**Published:** 2022-01-18

The above article from FEBS Open Bio, published online on 10 November 2015 in Wiley Online Library (wileyonlinelibrary.com), has been retracted by agreement between the corresponding author Zhenfan Qu, the journal's Editor‐in‐Chief Miguel A. De la Rosa and John Wiley & Sons Ltd. The retraction has been agreed following several concerns brought to the journal’s attention relating to duplicated elements between some of the figures in this article and those in an article published in FEBS Letters https://doi.org/10.1016/j.febslet.2014.11.031 and a duplication in an article published in OncoTargets and Therapy https://doi.org/10.2147/OTT.S87976, as described below:

1. The nontumour plot in Fig. 1A of the above paper is almost identical to Fig. 1A, NT in https://doi.org/10.2147/OTT.S87976, and this is not expected to have occurred by chance.
2. Table 1 in the above paper is almost identical to Table 1 in https://doi.org/10.1016/j.febslet.2014.11.031, with the exception of the values and the miRNA name.
3. In Fig. 2B of the above paper, the micrographs in the 2 bottom panels partially overlap with the corresponding panels of Fig. 3A in https://doi.org/10.1016/j.febslet.2014.11.031.
4. The graph in Fig. 4B of the above paper is identical to the graph in Fig. 4D of https://doi.org/10.1016/j.febslet.2014.11.031, with the exception of the names of the miRNAs.
5. There are duplicated regions in Fig. 5B of the above article.
6. There are duplicated bands between the blots in Fig. 5A of the above article and Fig. 6A of https://doi.org/10.1016/j.febslet.2014.11.031.

In addition, the same email address for correspondence was provided for the above article and for https://doi.org/10.1016/j.febslet.2014.11.031, but these two articles have different corresponding authors.

The paper has been retracted on the basis of the above duplications.
